# Cystathionine *β*-Synthase in Physiology and Cancer

**DOI:** 10.1155/2018/3205125

**Published:** 2018-06-28

**Authors:** Haoran Zhu, Shaun Blake, Keefe T. Chan, Richard B. Pearson, Jian Kang

**Affiliations:** ^1^Division of Research, Peter MacCallum Cancer Centre, 305 Grattan Street, Melbourne, Victoria 3000, Australia; ^2^Sir Peter MacCallum Department of Oncology, Australia; ^3^Department of Biochemistry and Molecular Biology, University of Melbourne, Parkville, Victoria 3052, Australia; ^4^Department of Biochemistry and Molecular Biology, Monash University, Clayton, Victoria 3168, Australia

## Abstract

Cystathionine *β*-synthase (CBS) regulates homocysteine metabolism and contributes to hydrogen sulfide (H_2_S) biosynthesis through which it plays multifunctional roles in the regulation of cellular energetics, redox status, DNA methylation, and protein modification. Inactivating mutations in CBS contribute to the pathogenesis of the autosomal recessive disease CBS-deficient homocystinuria. Recent studies demonstrating that CBS promotes colon and ovarian cancer growth in preclinical models highlight a newly identified oncogenic role for CBS. On the contrary, tumor-suppressive effects of CBS have been reported in other cancer types, suggesting context-dependent roles of CBS in tumor growth and progression. Here, we review the physiological functions of CBS, summarize the complexities regarding CBS research in oncology, and discuss the potential of CBS and its key metabolites, including homocysteine and H_2_S, as potential biomarkers for cancer diagnosis or therapeutic targets for cancer treatment.

## 1. Introduction

**Figure 1:**
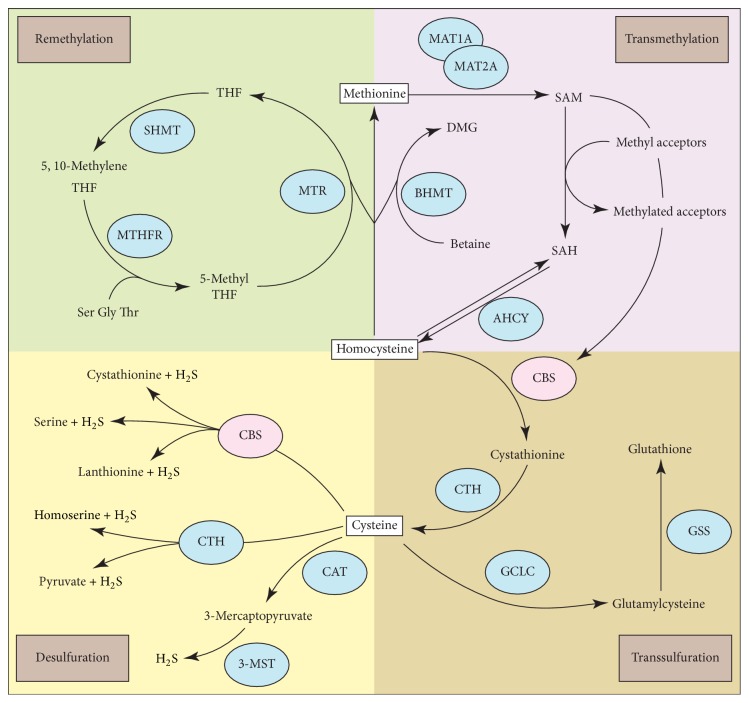
*Metabolic reactions catalyzed by CBS.* CBS catalyzes the condensation of homocysteine (Hcy) with serine to form cystathionine which is subsequently cleaved by cystathionine gamma-lyase (CTH) to form cysteine, a precursor of glutathione. CBS also catalyzes the production of H_2_S. In addition to CBS, CTH and 3-mercaptopyruvate sulfurtransferase (3-MST) are also involved in the conversion of cysteine to H_2_S. Homocysteine is another key CBS-derived metabolite and is linked to the metabolism of methionine. Methionine is converted to homocysteine via S-adenosyl methionine (SAM) and S-adenosyl homocysteine (SAH), releasing a methyl group that is used in numerous methylation reactions. SAM is an allosteric activator of CBS. 3-MST, 3-mercaptopyruvate sulfurtransferase; AHCY, adenosylhomocysteinase; BHMT, betaine-homocysteine methyltransferase; CAT, cysteine aminotransferase; CBS, cystathionine *β*-synthase; CTH, cystathionine gamma-lyase; GCLC, gamma-glutamylcysteine synthetase; GSS, glutathione synthetase; MAT1A/2A, methionine adenosyltransferase 1A/2A; MTHFR, methylenetetrahydrofolate reductase; MTR, 5-methyltetrahydrofolate-homocysteine methyltransferase; SAM, S-adenosyl methionine; SAH, S-adenosyl homocysteine; SHMT, serine hydroxymethyltransferase.

Cystathionine *β*-synthase (CBS) catalyzes the condensation of homocysteine (Hcy) with serine to form cystathionine, which is the initial and rate-limiting step in the transsulfuration pathway. Cystathionine is subsequently cleaved by the enzyme cystathionine gamma-lyase (CTH) to form cysteine, a precursor of glutathione. Besides this canonical pathway, CBS also participates in the desulfuration reactions that contribute to endogenous hydrogen sulfide (H_2_S) production (Figure 1). Thus, CBS acting mainly through control of Hcy and H_2_S metabolism exerts diverse biological functions including mitochondrial bioenergetics, redox homeostasis, DNA methylation and protein modification. Deregulation of CBS and the associated alterations in Hcy and/or H_2_S levels leads to a wide range of pathological disturbances in the cardiovascular, immune, and central nervous systems and contributes to disease development, such as CBS-deficient homocystinuria (CBSDH). It is now becoming clear that CBS activity also plays an important but complex role in cancer biology. This review focuses on the current understanding of the functional role of CBS and the derived metabolites Hcy and H_2_S in cancer pathogenesis and provides insight into the development of novel prognostic markers and therapeutic approaches for cancer patients.

## 2. CBS Protein Structure and Biological Functions

The human* CBS* gene encodes a protein of 551 amino acids. The crystal structure of the active form of human CBS, formed by four of 63-kDa subunits, has been fully characterized [1, 2]. Each subunit consists of three structural domains. The N-terminal domain binds to the cofactor heme, which is required for successful protein folding and assembly but not necessary for catalytic activity [3]. The catalytic domain encompasses a binding site for another cofactor, pyridoxal-phosphate (PLP) [4]. The C-terminal regulatory domain contains two CBS motifs (CBS1 and CBS2) that dimerize to form a Bateman domain. This domain is responsible for CBS subunit tetramerization and contains the binding sites for the allosteric activator S-adenosylmethionine (SAM) [1, 5, 6]. In the native quaternary structure, the access of substrates to the catalytic core is occluded by the C-terminal regulatory motifs and the binding of SAM induces a conformational change that improves the access of the substrates to the catalytic site [2]. The autoinhibitory function of the C-terminal regulatory domain is relieved by the C-terminal truncation that generates a 45 kDa isoform with higher basal catalytic activity than the full-length form [1].

CBS is predominantly expressed in the brain, liver, kidney, and pancreas. It is mainly a cytosolic enzyme, but localization in the nucleus [7] and mitochondria [8] had been detected in specific cell types. CBS can be translocated to the mitochondria in response to hypoxia [9] or nucleolar stress [10]. CBS expression is regulated at multiple levels upon different stimuli. For example, hormonal regulation by glucocorticoids increases CBS expression at the transcriptional level in liver cells, a process that may be perturbed by insulin administration through binding to an insulin-sensitive sequence localized on the CBS promoter [11]. In addition, testosterone can regulate CBS expression and activity in renal tissue [12]. Growth/differentiation factors such as EGF, TGF-*α*, cAMP, and dexamethasone induced CBS protein expression in mouse astrocytes [13]. Hypoxia upregulated CBS expression either via hypoxia-inducible factor- (HIF-) 1 at the transcriptional level [14] or decreased degradation of CBS protein by Lon proteases in the mitochondria [9]. Besides HIF-1, the zinc finger transcription factor SP1 binds to the* CBS* gene promoter, establishing its role as a key regulator of CBS expression [15, 16]. Furthermore, CBS activity may be enhanced via posttranslational regulation through S-glutathionylation [17] or inhibited via epigenetic downregulation of CBS expression through promoter methylation [18, 19].

CBS plays a critical role in Hcy elimination. Patients with CBS deficiency exhibit elevated Hcy plasma levels at excess of 200 *μ*M compared to 5-15 *μ*M in healthy adults [20]. CBS-deficient homocystinuria (CBSDH) is an autosomal recessive metabolic disease, resulting from inactivating mutations in the* CBS* gene. CBSDH patients present multiple pathologic changes in the eye, skeleton, central nervous, and vascular systems. Common symptoms in CBSDH patients include thrombosis, osteoporosis, and impaired mental cognitive development (reviewed in [21–23]). Administration of high doses of the PLP precursor, pyridoxine, or vitamin B_6_ is common treatment that ameliorates approximately 50% of clinical symptoms. To date, 164 pathogenic genetic variants have been identified (http://cbs.lf1.cuni.cz/mutations.php) of which the predominant mutations are missense mutations. c.833 T > C (p.I278T) is the most frequent mutation detected in many European populations [24]. The I278T missense mutation and many of the less prevalent mutations likely affect the folding or stability of the CBS protein [25] whereas some mutations such as mutant D444N, a missense mutation in the C-terminal regulatory domain, showed an approximately twofold increase in basal CBS activity but impaired response to SAM stimulation [2]. The pathophysiology of CBS deficiency is still not fully understood. As well as the accumulation of Hcy, CBS defects lead to increased concentrations of methionine and S-adenosyl-L-homocysteine (SAH) and depletion of cystathionine and cysteine. These perturbations may act in concert with high Hcy to promote the development and progression of CBSDH (reviewed in [26]).

Accordingly, extensive studies in the mouse models of CBS deficiency showed mice with homozygotic* CBS* deletion (CBS-/-) died within 4 weeks after birth due to severe hepatic dysfunction and exhibited extremely high levels of circulating Hcy (reviewed in [26, 27]). Wang et al. showed that the neonatal lethality could be rescued by decreasing circulating Hcy levels in a transgenic mouse model with inducible CBS expression [28]. They further found that there may be a threshold effect with Hcy, meaning that moderately lowering homocysteinemia can improve mouse viability during the neonatal period [29]. In support of the Hcy threshold effect, CBS+/- heterozygote mice were fully viable with a 3-fold increase of Hcy levels compared to the 8-fold increase in homozygous mice [30].

## 3. Homocysteine and H_2_S, the Major CBS-Derived Metabolites

### 3.1. Homocysteine

Hcy is a sulfur-containing nonproteinogenic amino acid linked to the metabolism of methionine and cysteine. Methionine is converted to Hcy via S-adenosyl methionine (SAM) and SAH, releasing a methyl group that is used in numerous methylation reactions. Hcy can reform Met by the remethylation pathway either via 5-methyltetrahydrofolate-homocysteine methyltransferase (MTR, 5-methyltetrahydrofolate as the methyl group donor) or betaine-homocysteine methyltransferase (BHMT, betaine as the methyl group donor) (Figure 1). Hcy is also irreversibly metabolized by CBS to cystathionine that subsequently converts to cysteine via CTH in the transsulfuration pathway (Figure 1). Hcy metabolism mainly occurs in the liver and conversion to cystathionine by CBS is a major elimination route of Hcy [31].

**Figure 2:**
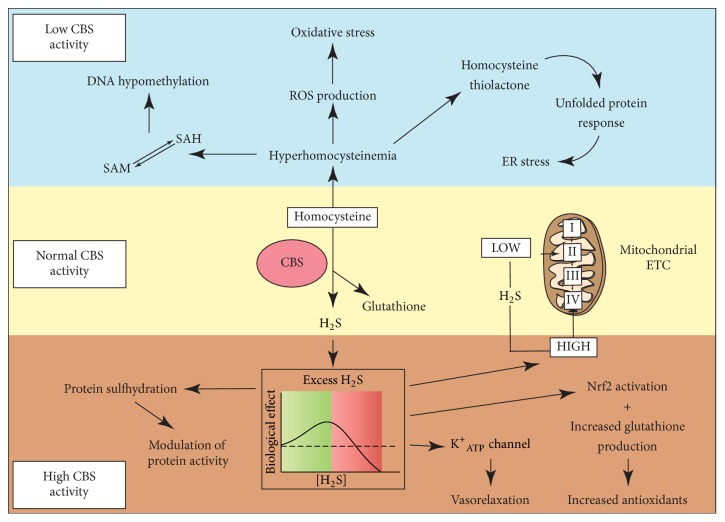
*Potential mechanisms underlying CBS deregulation with alterations of homocysteine and H*_2_ *S levels in cancer pathogenesis.* CBS deficiency causes hyperhomocysteinemia. Elevated Hcy concentration can increase reactive oxygen species (ROS) production and induce oxidative stress. Hyperhomocysteinemia also leads to formation of homocysteine thiolactone as a result of error-prone editing by the methionyl-tRNA synthase. This homocysteine derivative can cause protein N-homocysteinylation that impairs protein function, resulting in an unfolded protein response and endoplasmic reticulum (ER) stress. The elevated Hcy level can lead to accumulation of S-adenosyl homocysteine (SAH), a competitive inhibitor of most methyltransferases, consequently inducing DNA hypomethylation and affecting gene transcription. CBS-driven endogenous H_2_S production maintains mitochondrial respiration and ATP synthesis, promotes antioxidant production by enhancing Nrf2 activation and increasing glutathione production, and modulates protein activity via protein sulfhydration. Secreted H_2_S can cause vasodilation via activation of ATP-sensitive K^+^ channels.

Hyperhomocysteinemia (HHcy) is recognized as an independent risk factor for atherosclerotic vascular disease [32]. HHcy may result from mutations in genes encoding enzymes of Hcy biosynthesis and metabolism or deficiencies of vitamin cofactors including vitamin B_12_ and B_6_ [33]. The molecular mechanisms underlying HHcy-induced atherosclerosis are complex and multifactorial (Figure 2). Elevated Hcy concentration reduces nitric oxide (NO) bioavailability and causes oxidative stress. HHcy also leads to formation of Hcy thiolactone as a result of error-prone editing by the methionyl-tRNA synthase [34]. This Hcy derivative can cause protein N-homocysteinylation in which the thioester group of thiolactone binds to the lysine residues in proteins, consequently impairing protein function, resulting in unfolded protein response and endoplasmic reticulum stress (reviewed in [35, 36]). Moreover, an elevated Hcy level could lead to accumulation of SAH, a competitive inhibitor of most methyltransferases, consequently inducing DNA hypomethylation [37]. Through this epigenetic mechanism, Hcy has been reported to inhibit endothelial cell growth by decreasing the expression of cyclin A [38], fibroblast growth factor 2 [39], and hTERT expression [40] and by upregulation of platelet-derived growth factors and P66shC [41].

HHcy has also been implicated in the pathogenesis of cancer. Increased release of Hcy by tumor cells is related to their rapid proliferation rate [42]. Hcy accumulation results from defects in methionine synthesis, leading to a methionine-dependent malignant phenotype [43]. A meta-analysis revealed the association of elevated circulating Hcy levels with increased overall risk of cancer [44]. A higher Hcy plasma level has been detected in the patients with hepatocellular carcinoma (HCC) [44] and head and neck squamous cell carcinoma [45]. Although the mechanisms underlying this association between elevated Hcy levels and malignant transformation are unclear, a recent study proposed a mechanism linking Hcy to lipid metabolism and HCC [46]. It demonstrated that Hcy transcriptionally upregulated CYP2J2, a cytochrome P450 (CYP) epoxygenase by stimulating DNA demethylation and increasing SP1/AP1 activity on the promoter of CYP2J2, which promotes epoxyeicosatrienoic acid synthesis and hepatocellular tumorigenesis.

### 3.2. H_2_S

Like nitric oxide and carbon monoxide, H_2_S is a diffusible gaseous transmitter in the human body and is mainly synthesized during cysteine metabolism and excreted as urinary sulfates by the kidney (reviewed in [47]). CBS catalyzes the production of H_2_S via at least three pathways including (i) converting cysteine to serine and H_2_S, (ii) condensing cysteine and Hcy to yield cystathionine and H_2_S, and (iii) condensing two cysteine molecules to lanthionine and H_2_S (Figure 1). In addition to CBS, CTH and 3-mercaptopyruvate sulfurtransferase (3-MST) are also involved in the conversion of cysteine to H_2_S (Figure 1).

While H_2_S has diverse biological functions in the nervous, cardiovascular, and immune systems, the pathological role of H_2_S in cancer biology has attracted substantial attention in recent years. CBS-driven endogenous H_2_S production has been reported to support tumor growth by (i) maintaining mitochondrial respiration and ATP synthesis, (ii) stimulating cell proliferation and survival, (iii) redox balance, and (iv) vasodilation (Figure 2). H_2_S modulates mitochondrial functions and cellular bioenergetics in a concentration-dependent manner. At low concentrations, H_2_S acts as a mitochondrial electron donor to mitochondrial complex II, resulting in bioenergetic stimulation [48, 49]. At higher concentrations, H_2_S acts as a mitochondrial poison via the inhibition of cytochrome* c* oxidase in mitochondrial complex IV [50]. H_2_S stimulates cell proliferation through activation of specific kinase pathways (e.g., MAPK and PI3K/Akt) and inhibition of selective phosphatases such as PTEN and PTP1B [51–53]. Modulation of protein activity by H_2_S either occurs via protein sulfhydration (reviewed in [54]) or intracellular formation of polysulfides by H_2_S followed by oxidative inactivation of proteins [55, 56]. The sulfhydration of nuclear factor kappa B (NF-*κ*B) by H_2_S has also been shown to inhibit apoptosis and may be of particular relevance to cancer cell survival [57]. The protective effect of H_2_S from oxidative stress has been extensively studied in endothelial cells and neurons [58–62]. Studies showed H_2_S inhibited H_2_O_2_-mediated mitochondrial dysfunction by preserving the protein expression levels and activity of key antioxidant enzymes, inhibiting reactive oxygen species (ROS) production and lipid peroxidation [60]. Additionally, these effects may be associated with sulfhydration of Keap1 and activation of Nrf2 [61] or increasing the production of the antioxidant glutathione. Vasorelaxation is one of the first recognized biological effects of H_2_S. The mechanisms of H_2_S-mediated vasodilation include the activation of ATP-sensitive K^+^ channels, inhibition of phosphodiesterases, and a synergy with NO (reviewed in [63]).

H_2_S-donating compounds deliver H_2_S exogenously, including fast H_2_S donors such as sulfate salts (e.g., NaHS and Na_2_S) and naturally occurring compounds (e.g., the garlic constituent diallyl trisulfide, sulforaphane, erucin, and iberin) and slow H_2_S-releasing synthetic moieties such as GYY4137 (reviewed in [64]). The cellular response to exogenous H_2_S released by the donors has been considered as a biphasic response, in which low H_2_S concentrations (or low H_2_S production rates) showed enhancement of cell proliferation rates and cell viability whereas high H_2_S caused deleterious/adverse effects in cells [50, 65]. This biphasic cellular response is consistent with the special action model of H_2_S on mitochondrial respiration described above, that is, stimulation of mitochondrial respiration at low levels and inhibition at high levels. This bell-shape pharmacology of H_2_S may, at least in part, explain the inconsistent results of the effect of exogenous H_2_S in colon cancer cell line HCT116 reported by different groups including a growth inhibitory effect (using NaHS at 400 *μ*M and 800 *μ*M) by the Deng lab [66] and a growth stimulatory effect (using NaHS at 30-300 *μ*M) by the Szabo lab [49, 65, 67].

## 4. CBS and Cancer

### 4.1. Promoting Tumor Growth by Activation of CBS

Elevated expression of CBS in tumor tissues or cell lines has been reported in colon [49, 68], ovarian [8], prostate [69], and breast cancer [70], compared to adjacent normal tissue or nontransformed cells. A series of studies from the Hellmich group characterized the oncogenic role of CBS in colon cancer [49, 68, 71]. Through modification of CBS expression (overexpression or RNAi knockdown) or CBS activity (allosteric activator SAM or the inhibitor aminooxyacetate) in the HCT116 colon cancer cell line, they demonstrated that CBS promoted cancer cell proliferation. The antiproliferative effect observed by silencing or inhibiting CBS was recapitulated in the xenograft mouse models and patient-derived tumor xenografts [49]. CBS not only promotes tumor growth and progression but also initiates tumor formation [68]. Overexpression of CBS in adenoma-like colonic epithelial cell line NCM356 enhanced cell proliferative, anchorage-independent growth and invasive capability* in vitro* and tumorigenicity* in vivo*. Mice heterozygous for CBS showed fewer numbers of mutagen-induced aberrant crypt foci than wild-type controls. Through a similar approach, Bhattacharyya et al. [8] reported that CBS knockdown inhibited cell proliferation and suppressed tumor growth in an orthotopic mouse model of cisplatin-resistant ovarian cancer. Interestingly, in breast cancer silencing CBS did not affect cell proliferation in culture but significantly attenuated tumor growth in a xenograft mouse model [70].

The protumorigenic effect of CBS occurs through an autocrine mechanism by regulation of bioenergetics, antioxidant capacity, and apoptosis-related pathways. Targeting CBS genetically or pharmacologically impairs cellular bioenergetics through inhibiting mitochondrial electron transport, oxidative phosphorylation, and glycolysis. H_2_S was identified to be responsible for such metabolic and bioenergetic rewiring in colon cancer cells, as CBS expression and activity correlated with H_2_S production and exogenous H_2_S stimulated cell proliferation and bioenergetics [49]. Systematic metabolomic analysis of CBS-overexpressing NCM356 cells uncovered an anabolic metabolic phenotype with significantly enhanced glycolysis, nucleotide synthesis, and lipogenesis, which is thought to promote malignant transformation [68]. CBS may also promote tumor cell survival by increasing cell intrinsic antioxidant capacity. Ovarian cancer cells depleted of CBS showed enhanced ROS production. Antioxidant glutathione, but not H_2_S, fully rescued viability of CBS-depleted cells, suggesting that the effect of CBS in ovarian cancer cells is mediated through regulation of ROS production by glutathione [8]. Similarly, reduced glutathione abundance was observed in breast cancer cells upon CBS silencing and was accompanied by decreased Nrf2 expression [72]. CBS downregulation reduced antioxidant capacity and enhanced the sensitivity of cancer cells to chemotherapeutic drugs. The cytoprotective effect of CBS is also associated with regulation of NF-*κ*B and p53 apoptosis-related signaling [8]. A recent study further suggested CBS is involved in nucleolar stress-induced apoptosis [10]. The authors demonstrated that treatment of p53-/- colon cancer cells with 5-fluorouracil caused nucleolar stress, which led to accumulation of the ribosome-free form of ribosomal protein L3 (rpL3). rpL3 decreased CBS protein abundance through suppression of SP1-mediated* CBS* gene transcription and increase of CBS protein degradation by translocation of CBS into mitochondria. Decreased CBS abundance and, in turn, reduction of H_2_S production have been suggested to contribute to mitochondrial cytochrome C release and induction of the intrinsic cell death pathway [10].

In addition to autocrine regulation, CBS acts via a paracrine mechanism to modulate the tumor microenvironment including stimulating angiogenesis and vasodilation via H_2_S production and release as reported in colon and ovarian cancer xenografts [8, 49] and regulating macrophage activation in breast cancer xenograft mouse models [70].

### 4.2. CBS Associated Oncogenesis Is Tumor Type-Specific

Unlike in colon, ovarian, and breast cancer, CBS does not appear to have a functional role in melanoma [73]. CBS expression is absent in dysplastic nevi, detected in only 25% of primary melanoma samples, and unregulated in four of five melanoma cell lines examined. More importantly, modulation of CBS expression had a minimal effect on melanoma cell proliferation [73].

**Figure 3:**
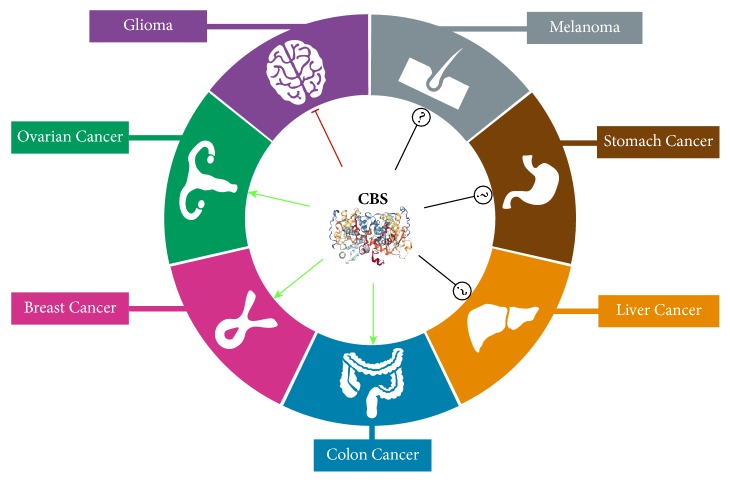
*CBS associated oncogenesis is tumor type-specific.* Activation of CBS promotes tumor growth in colon, ovarian, and breast cancer but suppresses tumor growth in glioma. The role of CBS in liver cancer, gastric cancer, and melanoma is still conflicting and inconclusive.

Downregulation of CBS through promoter methylation has been observed in multiple gastric cancer cell lines and four colon cancer cell lines (including HCT116) [74]. However, the biological consequence of CBS epigenetic silencing in gastric cancer has not been determined. Evidence from glioma supports a tumor-suppressive role for CBS [75]. CBS deficiency in U87-MG glioma cells did not affect cell proliferation in 2D culture but increased colony formation in soft agar, indicative of enhanced anchorage-independent growth. Consistently, CBS knockdown decreased tumor latency in U87-MG xenografts and increased tumor volume in an orthotopic model. Enhanced glioma tumorigenicity upon CBS loss was associated with upregulation of HIF-2*α* protein level and HIF-2*α*-dependent transcriptional activation of angiopoietin like 4 (ANGPTL4) and vascular endothelial growth factor A (VEGFA). The lack of function or suppression of tumor growth by CBS in certain tumor types indicates that CBS associated oncogenesis is tumor-specific (Figure 3).

### 4.3. Conflicting Role of CBS in Hepatocellular Carcinoma

Clinical evidence from patient samples strongly supports a negative regulatory role for CBS in hepatocellular carcinoma (HCC). Downregulation of CBS expression and activity contributes to the pathogenesis of multiple liver diseases (Reviewed in [76]). Analysis of 120 HCC specimens found that CBS mRNA was markedly lower in tumor tissues than surrounding noncancerous liver [77]. Reduced CBS expression was significantly correlated with the poor clinic pathological parameters including tumor stage, Edmondson grade, alpha-fetoprotein (AFP) level, and overall survival. Further data analysis suggested that the expression level of CBS mRNA could be used as a prognostic marker for overall survival especially in patients with low AFP levels [77]. Diminished CBS levels were also detected in the tumor tissues from the mouse model of HCC [78–80]. Further supporting the tumor-suppressive role for CBS, exogenous H_2_S induced autophagy and apoptosis in HCC cells through the PI3K/Akt/mTOR pathway [81].

Intriguingly, distinct from this clinical data, a recent study showed that several HCC cell lines exhibited higher CBS expression than normal liver cells HL-7702 and QSG-7701 [82]. Both genetic (by siRNA) and pharmacological (by AOAA) inhibition of CBS in the SMMC-7721 HCC cell line with reduced H_2_S production decreased cell viability and enhanced ROS production in* vitro*. Another study showing that the PI3K/AKT pathway regulated the CTH/H_2_S to promote HCC proliferation also supports the oncogenic role of H_2_S in HCC [53]. Clearly, the biological function of CBS in liver cancer is complex and requires further investigation.

## 5. CBS in Cancer Therapy

Consistent with the complex roles of CBS in cancer biology described above, it is also becoming evident that both the activators and inhibitors of CBS have antitumor activity in different cancer models. This genetic context dependence determines different types of cancer will display distinct efficacy and toxicity profiles in response to CBS-based targeted therapies.

### 5.1. CBS Inhibitors

Aminooxyacetate (AOAA) is currently considered as the most potent CBS inhibitor compared with the other drugs such as trifluoroalanine and hydroxylamine [65]. It has shown antitumor actions in the mouse xenograft models of colon [49] and breast cancer [83] and patient-derived colon cancer xenografts [49]. Decreased H_2_S level in plasma was detected in a colon xenograft mouse model treated with AOAA while the drug effect on circulating Hcy level was not investigated. While these antitumor responses are encouraging, the therapeutic effect of CBS inhibition requires further investigation as AOAA is actually not selective for CBS [65, 84]. The pharmacological action of AOAA is not limited to suppression of the CBS/ H_2_S axis. It binds irreversibly to the cofactor PLP, and therefore, in addition to CBS, it inhibits other PLP-dependent enzymes such as CTH, 3-MST, and glutamate oxaloacetate transaminase 1 (GOT1). AOAA has been reported to target CTH preferentially over CBS (IC50 8.52 *μ*M for CBS versus 1.09 *μ*M for CTH) [85]. Furthermore, inhibition of GOT1 by AOAA disrupted the malate/aspartate shuttle, decreased glucose-derived carbon flux into mitochondrial tricarboxylic acid cycle, and ATP synthesis [83].

To identify new CBS inhibitors, two groups performed small-molecule screening [86, 87]. The Barrios group [87] and the Wu group [86] used recombinant CBS enzymes and employed fluorescent H_2_S readouts to screen a composite library of 1900 compounds and a chemical library consisting of 20,000 compounds, respectively. Several compounds showed some selectivity for CBS compared with CTH with IC50 20-50 *μ*M. However, as the studies did not use AOAA as a reference in the screen, whether these drugs are superior to AOAA in terms of potency and selectivity remains unknown.

### 5.2. CBS Activator S-Adenosyl-L-Methionine (SAM)

SAM is a vital molecule for transmethylation and transsulfuration reactions. It is the principle methyl-donor for DNA, amino acid, protein, and lipid methyltransferase and a key precursor for glutathione and polyamine synthesis (reviewed by [88]). It is synthesized from methionine and ATP by methionine adenosyltransferase (MAT, Figure 1). SAM, as an allosteric activator, modulates CBS activity by inducing a conformational change in the C-terminus of CBS that facilitates the entrance of substrates into the catalytic site of the enzyme [1]. Although SAM has been used for treatment of osteoarthritis [89], depression [90], and liver diseases [88], the clinical evidence for its efficacy in these diseases is still inconclusive. Recent data support the concept of using SAM as a chemopreventive agent in HCC and colon cancer, consistent with the proposed tumor-suppressive role of CBS in HCC. The* Mat1a* knockout mice spontaneously develop HCC supporting the fact that hepatic SAM deficiency predisposes to HCC [91]. In several rodent models of HCC, administration of SAM is effective in preventing liver carcinogenesis [92, 93]. One phase II clinical trial is evaluating SAM as a potential chemoprevention agent in patients with hepatitis C cirrhosis [94]. SAM also showed a similar chemoprevention effect in an inflammation induced colon cancer mouse model [95]. In addition to chemoprevention, SAM exerted a proapoptotic effect in liver (at 0.2 mM over 5 days) [96], gastric (10 *μ*M over 7 days) [97], and colon cancer cells (ranging from 0.25 to 5 mM for 24 hours) [98]. Interestingly, similar to the conflicting data regarding CBS function and effects of H_2_S donors in colon cancer, the Szabo group [71] reported a biphasic response to SAM in colon cancer cells. At low concentrations for the short-time period (0.1-1 mM for 12 hours or 0.1 mM for 24 hours), SAM induced a stimulatory effect on CBS activation, H_2_S production, and cell proliferation, while at higher concentrations or chronic exposure (0.1-5 mM after 24 hours) the inhibitory effects became more prominent and were not attenuated by CBS silencing, suggesting nonspecificity or toxicity [71]. Therefore, more work in multiple experiment models is required to better define the role of SAM/CBS axis in cancer pathogenesis.

## 6. CBS in Cancer Prognosis

With the identification of the pathogenic role of CBS in cancer, the use of CBS as a prognostic and predictive biomarker is becoming attractive. As described above, the negative correlation of CBS expression with the pathologic parameters in HCC indicates its potential as a prognostic marker in HCC [77]. Modulation of CBS activity can be indicated by the changes of Hcy and/or H_2_S levels. The potential prognostic values of Hcy in cancer have been extensively studied [99–101]. However, the biological sources of Hcy were not defined in these studies and, thus, the link between the levels of Hcy and CBS function remains unknown. Nevertheless, significant progress in the detection and quantitation of Hcy from patient samples has been made in recent years. Methods of measuring plasma Hcy have evolved from ion-exchange chromatography to high-performance liquid chromatography (HPLC), gas-chromatography mass spectrometry, liquid chromatography-electrospray tandem mass spectrometry (LC-MS/MS), and fluorescence polarization immunoassay (FPIA) [102]. In terms of H_2_S, elevated H_2_S in exhaled breath or its degraded form in urine in cancer patients provides support for the clinical utility of H_2_S as a marker of cancer [101]. However, in order to determine the prognostic and predictive values of H_2_S in cancer, development of the methods that can accurately measure H_2_S levels in the circulation or in the targeted organs is imperative.

## 7. Summary and Future Directions

A functional role for CBS in tumor biology is supported by (i) clinical evidence of altered CBS expression level and CBS-derived Hcy and H_2_S levels in cancer patients; (ii) preclinical studies showing dysregulation of CBS function and activity in cancer cell culture and animal models; (iii) mechanistic investigations linking CBS to cancer-related cellular and molecular changes and signaling pathways. The distinct biological effects of CBS alterations in different cancer models reveal the complexity of CBS signaling in cancer pathogenesis. The contradictory role of CBS in cancer biology (Figure 3) is possibly due to the existence of alternative Hcy and H_2_S metabolic pathways, and multiple modes of regulation of CBS expression and activity by hormones, growth factors, and other metabolites. Therefore, the functional role of CBS is determined by the distinct metabolic and genetic profiles in different types of cancer and is context-dependent. Furthermore, the current conflicting data adds an additional layer of complexity, indicating that multiple experimental and analytical approaches as well as in-depth mechanistic investigations are required to clarify the role of CBS in cancer biology.

Increased understanding of the role of the CBS-controlled network in cancer biology will greatly promote the development of pharmacological reagents targeting CBS and the identification of appropriate patient populations. CBS acts through two main metabolites Hcy and H_2_S, which have important physiological roles in specific tissues such as the liver, brain, and blood vessels. Given its central metabolic role, it is possible that CBS-based targeted therapy may cause side effects due to accumulation of unfavorable metabolites. For example, CBS inhibitors may elevate Hcy levels with potential risk for developing HHcy. Therefore, further studies will be required to define the therapeutic windows of the novel CBS targeting agents. Additional investigations are clearly required to better elucidate the complex role of CBS in malignant transformation including (i) characterizing the role of CBS-related metabolic signaling in cancer pathogenesis including but not limited to CBS, Hcy, H_2_S, and the related enzymes; (ii) determining the interaction of tumor cell-derived CBS and its metabolites with the microenvironment; (iii) identifying biomarkers of CBS-based therapies in clinical samples and cancer models. Certainly, a greater appreciation for the complexity of CBS in cancer biology will give rise to new prospective biomarkers or targets for cancer.
